# The prognostic value of negative lymph node count for patients with cervical cancer after radical surgery

**DOI:** 10.18632/oncotarget.23596

**Published:** 2017-12-21

**Authors:** Hao Lu, Rong Guo, Haotian Yang, Haolu Wang, Xiaowen Liang, Zhiqian Hu, Xinxing Li

**Affiliations:** ^1^ Department of General Surgery, Changzheng Hospital, The Second Military Medical University, Shanghai, China; ^2^ The First Affiliated Hospital of Gannan Medical University, Ganzhou, Jiangxi, China; ^3^ Therapeutics Research Centre, The University of Queensland Diamantina Institute, The University of Queensland, Translational Research Institute, Woolloongabba, QLD, Australia; ^4^ School of Biomedical Sciences, The University of Queensland, St Lucia, QLD, Australia

**Keywords:** negative lymph node, cervical cancer, radical surgery, SEER

## Abstract

Negative lymph node (NLN) count has been recognized as a prognostic indicator in various cancers. However, the relationship between NLN count and the prognosis of cervical cancer is still unknown. In this study, 10, 500 cervical cancer patients after radical surgery were selected from Epidemiology and End Results Program (SEER) data. Clinicopathological characteristics were collected for analysis, including year of diagnosis, age, race, grade, primary site, FIGO stage and cause specific survival (CSS). Univariate and multivariate Cox proportional hazards model was used to assess risk factors for survival of patients. X-tile plots identified 6 as the optimal cutoff value of NLN count to divide patients into high and low risk subsets in terms of CSS (χ^2^ = 183.95, *P* < 0.001). The rate of 5-year CCS of cervical cancer patients was improved with an increase in NLN count from 0 to 23 (all *P* < 0.001). NLN count was validated as an independently prognostic factor by the multivariate Cox analysis (HR: 1.571, 95% CI: 1.370~1.801, *P* < 0.001). Subgroup analysis showed that NLN count was a prognosis factor in FIGO stage I (χ^2^=35.023, *P* < 0.001), stage II (χ^2^ = 12.910, *P* < 0.001), stage III + IV (χ^2^ = 9.732, *P* = 0.002) and unknown stage (χ^2^ = 16.654, *P* < 0.001). Conclusively, this study demonstrated the NLN count was an independent prognostic factor for cervical cancer patients.

## INTRODUCTION

Cervical cancer is the fourth most common female malignancy worldwide [1–4]. For women with early-stage disease, radical hysterectomy and pelvic lymphadenectomy are the standard treatments and regional lymph node dissection is important to the survival outcome. Despite the lymph node status does not affect the staging of cervical cancer in the current International Federation of Gynecology and Obstetrics (FIGO) staging system [5], recent studies have revealed that the lymph node status such as lymph node ratio have prognostic value for survival of patients with cervical cancer [6–11]. The node-positive cervical cancer is heterogeneous and the prognosis of these patients cannot be stratified by the definite node-stage. The concept of negative lymph node (NLN) counts has attracted attention recently. NLN count can serve as a prognostic indicator in various cancers, such as colon [12], breast [13], esophagus [14] and gastric [15]. However, the correlation between NLN count and patient prognosis in cervical cancer is not fully studied. This retrospective study investigated the relationship between NLN counts and survival of cervical cancer patients who received radical surgery. We used Surveillance Epidemiology and End Results (SEER) database to investigate this association, and determine the optimal cutoff value of NLN counts.

## RESULTS

### Demographic and clinicpathological characteristics in SEER database

In total, we selected 10, 500 eligible patients with M0 cervical cancer who received radical surgery from 2004 to 2012. All patients did not receive neoadjuvant therapy. The median age of patients was 43 years (mean, 44.80 ± 12.91 years). The median survival time was 54 months in SEER-data. There were 1, 135 patients with NLN counts ranging from 0 to 6, and 9, 365 patients with NLN counts 7 or more than 7. These were 69.93% (7, 343/10, 500), 9.55% (1, 003/10, 500), 2.49% (261/10, 500), 0.30% (32/10, 500) and 17.72% (1, 861/10, 500) patients in FIGOI, II, III, IV and unknown stage, respectively. Since minimal cases in FIGO III and IV, we combined data of FIGO III and IV in the subsequent data analysis.

The demographic and tumor characteristics of patients were summarized in Table 1. A total of 31.15% (3, 271/10, 500) patients received postoperative radiotherapy. The NLN number was correlated with the year of diagnosis, age, race, grade, histologic type, FIGO stage and radiation after surgery (all *P* < 0.05).

**Table 1:** Baseline demographic and tumor characteristics of patients with cervical cancer in SEER database

Parameter	Characteristic	*N*	NLN = 0~6	NLN = 7~	χ^2^	*P* value
Year of diagnosis					9.597	0.002
	2004–2008	6656	672	5984		
	2009–2012	3844	463	3381		
Age					60.255	0.000
	<60	9236	918	8318		
	≥60	1264	217	1047		
Race					56.815	0.000
	White	8389	829	7560		
	Black	956	170	786		
	Others	1155	136	1019		
Grade					20.861	0.000
	I/II	4955	466	4489		
	III/IV	3394	395	2999		
	Unknown	2151	274	1877		
Primary Site					0.649	0.885
	Endocervix	2377	256	2121		
	Exocervix	338	33	305		
	Overlapping lesion	343	40	303		
	Cervix uteri	7442	806	6636		
Histologic type					9.019	0.011
	Squamous cell carcinoma	7377	841	6536		
	Adenocarcinoma	2901	274	2627		
	Mucinous adenocarcinoma	222	20	202		
FIGO stage					256.214	0.000
	I	7343	644	6699		
	II	1003	184	819		
	III	261	89	172		
	IV	32	11	21		
	Unknown	1861	207	1654		
Radiation					168.765	0.000
	No radiation	7299	590	6639		
	Radiation after surgery	3271	545	2726		

### The optimal cutoff points for NLNs determined by X-tile program

We analyzed the prognostic outcome utilizing various NLN count ranging from 0 to 23 to assess the impact of different NLN count on CSS. The 5-year CSS was calculated for patients with NLNs number or more nodes and less than NLN nodes. Table 2 demonstrated that NLN count was a prognosis factor (all *P <* 0.001). The 5-year CSS rate increased from 40.8% to 91.4%.

**Table 2:** Univariate analysis of the influence of different NLN count on CSS in patients with cervical cancer

NLN	No.	5-year CCS	χ^2^	*P* value	NLN	No.	5-year CCS	χ^2^	*P* value
≤0	49	40.8%	160.499	0.000	≤12	3143	85.1%	80.909	0.000
>0	10451	89.6%			>12	7357	91.2%		
≤1	173	60.0%	173.818	0.000	≤13	3545	86.1%	57.158	0.000
>1	10327	89.8%			>13	6955	91.0%		
≤2	349	68.4%	169.186	0.000	≤14	3937	86.5%	55.397	0.000
>2	10151	90.1%			>14	6563	91.1%		
≤3	482	70.4%	214.406	0.000	≤15	4323	86.8%	51.082	0.000
>3	10018	90.3%			>15	6177	91.2%		
≤4	670	73.4%	220.302	0.000	≤16	4718	87.0%	50.894	0.000
>4	9830	90.4%			>16	5782	91.3%		
≤5	867	75.4%	213.032	0.000	≤17	5093	87.3%	40.925	0.000
>5	9633	90.6%			>17	5407	91.3%		
≤6	1135	78.3%	182.842	0.000	≤18	5444	87.5%	44.011	0.000
>6	9365	90.7%			>18	5056	91.3%		
≤7	1404	80.2%	149.141	0.000	≤19	5870	87.9%	34.635	0.000
>7	9096	90.8%			>19	4630	91.2%		
≤8	1709	81.7%	123.485	0.000	≤20	6202	88.0%	32.770	0.000
>8	8791	90.8%			>20	4298	91.3%		
≤9	2059	83.2%	100.117	0.000	≤21	6536	88.1%	28.925	0.000
>9	8441	90.9%			>21	3964	91.4%		
≤10	2396	84.1%	90.895	0.000	≤22	6872	88.3%	24.252	0.000
>10	8104	90.9%			>22	3628	91.3%		
≤11	2761	84.4%	91.926	0.000	≤23	7141	88.4%	22.362	0.000
>11	7739	91.1%			>23	3359	91.4%		

**Figure 1:**
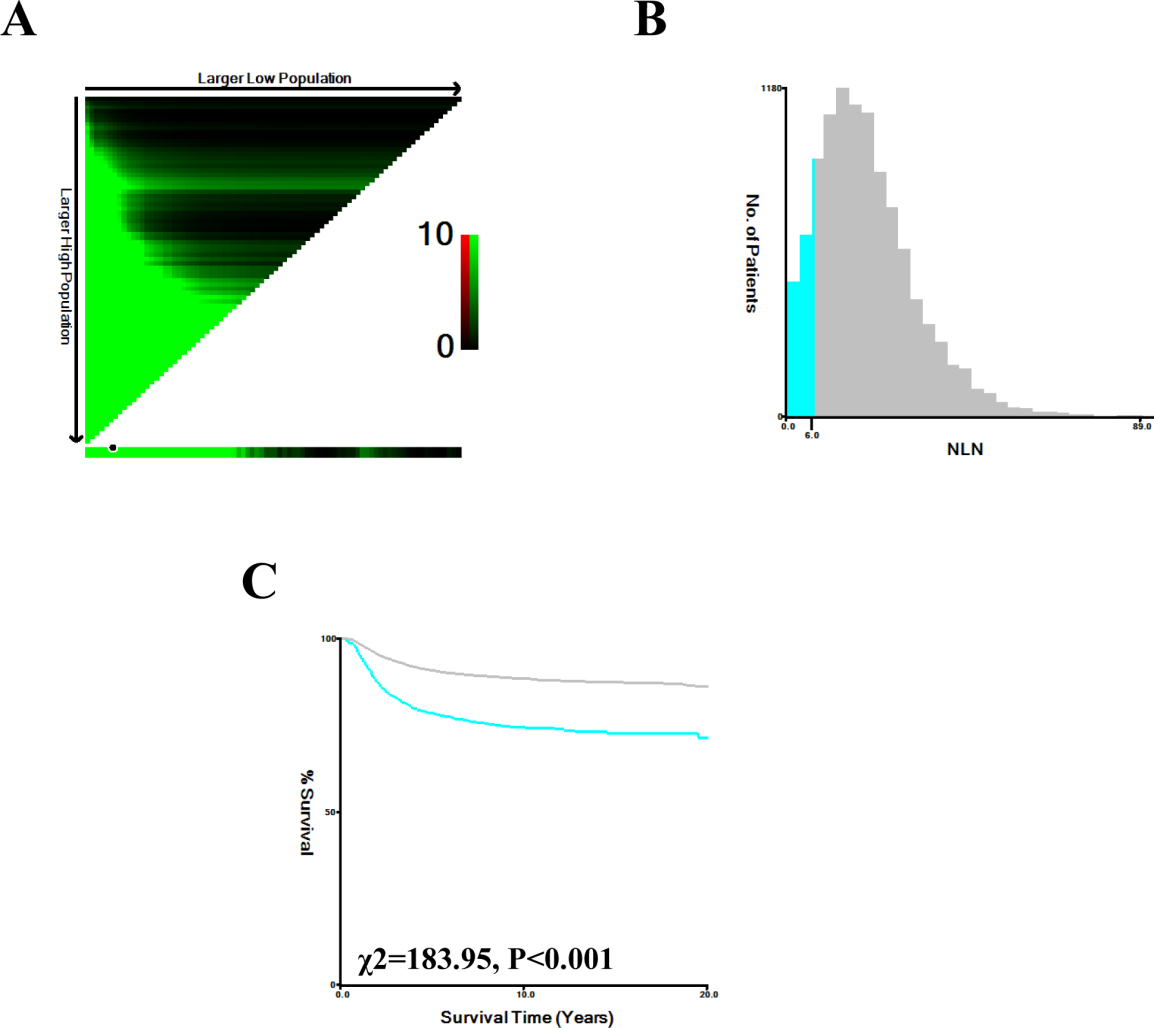
X-tile analysis of survival data from the SEER registry X-tile analysis was performed using patient data, equally divided into training and validation sets, from the SEER registry. X-tile plots of the training sets are shown in the left panels, with plots of matched validation sets shown in the smaller inset. The optimal cut-point highlighted by the black circle in the left panels is shown on a histogram of the entire cohort (middle panels), and a Kaplan-Meier plot (right panels). *P* values were determined using the cutoff point defined in the training set and applying it to the validation set. (The optimal cutoff value for NLN count is 6, χ^2^ = 183.95, *P* < 0.001).

As shown in Figure 1, X-tile plots were constructed and the maximum χ^2^ log-rank value of 182.842 (6 as the NLN count, *P <* 0.001) was produced, applying 6 as the optimal cutoff value to divide the patients into high and low risk subsets in terms of CSS. Compared to patients with NLN count ≤6, patients with NLN count >6 showed a significant improvement in 3 and 5-year CSS of 11.30% and 12.60%, respectively (Table 3).

**Table 3:** Univariate and multivariate survival analysis of cervical cancer patients who received radical surgery

Parameter	Characteristic	3-year CCS	5-year CCS	Univariate analysis		Multivariate analysis	
				Log rank χ^2^ test	*P*	HR (95%CI)	*P*
Year of diagnosis				1.743	0.187	NI	
	2004–2008	88.1%	83.5%				
	2009–2012	88.9%	82.3%				
Age				77.726	0.000		0.002
	<60	89.1%	84.2%			Ref	
	≥60	83.1%	74.5%			0.805 (0.699~0.926)	0.002
Race				12.908	0.002		0.009
	White	88.8%	83.6%			Ref	
	Black	85.8%	79.1%			1.064 (0.895~1.265)	0.482
	Others	87..8%	82.3%			1.350 (1.082~1.685)	0.008
Grade				156.785	0.000		0.000
	I/II	90.6%	85.5%			Ref	
	III/IV	83.0%	76.6%			1.242 (1.037~1.488)	0.019
	Unknown	91.9%	87.4%			1.966 (1.645~2.349)	0.000
Primary Site				24.410	0.000		0.266
	Endocervix	91.5%	86.2%			Ref	
	Exocervix	87.9%	84.3%			0.858 (0.732~1.006)	0.059
	Overlapping lesion	87.8%	83.1%			0.987 (0.719~1.354)	0.934
	Cervix uteri	87.4%	82.0%			0.881 (0.646~1.200)	0.421
Histologic type				70.867	0.000		0.000
	Squamous cell carcinoma	87.0%	81.3%			Ref	
	Adenocarcinoma	92.2%	87.9%			0.473 (0.353~0.633)	0.000
	Mucinous adenocarcinoma	84.7%	76.1%			0.544 0.404~0.733)	0.000
FIGO stage				934.468	0.000		0.000
	I	92.4%	88.0%			Ref	
	II	73.7%	64.7%			0.647 (0.560~0.748)	0.000
	III	58.2%	48.7%			1.406 (1.192~1.658)	0.000
	IV	21.8%	15.6%			2.367 (1.908~2.937)	0.000
	Unknown	85.9%	79.2%			6.989 (4.644~10.518)	0.000
Radiation				608.573	0.000		0.000
	No radiation	92.9%	89.0%			Ref	
	Radiation after surgery	78.4%	69.9%			0.321 (0.282~0.365)	0.000
NLN				127.645	0.000		0.000
	0~6	78.3%	71.8%			Ref	
	7~	89.6%	84.4%			1.571 (1.370~1.801)	0.000

NI: not included in the multivariate survival analysis.

### Impact of the number of NLNs on CSS in the patients with cervical cancer

Univariate analysis revealed that the number of NLNs (*P <* 0.001) and other clinicopathological factors, including age (*P* < 0.001), race (*P* = 0.002), grade (*P* < 0.001), primary site (*P <* 0.001), histologic type (*P <* 0.001), FIGO stage (*P* < 0.001), and radiation after surgery (*P <* 0.001) were significantly correlated with the prognostic outcome in cervical cancer patients (Table 3). According to the Multivariate Cox regression analysis, survival of cervical patients was improved (HR = 1.571, 95% CI: 1.370~1.801, *P <* 0.001) with an increase in the number of NLNs, indicating the number of NLNs was an independent predictors of CSS (Table 3).

### Impact of the NLN count on CSS in different FIGO stages

**Figure 2:**
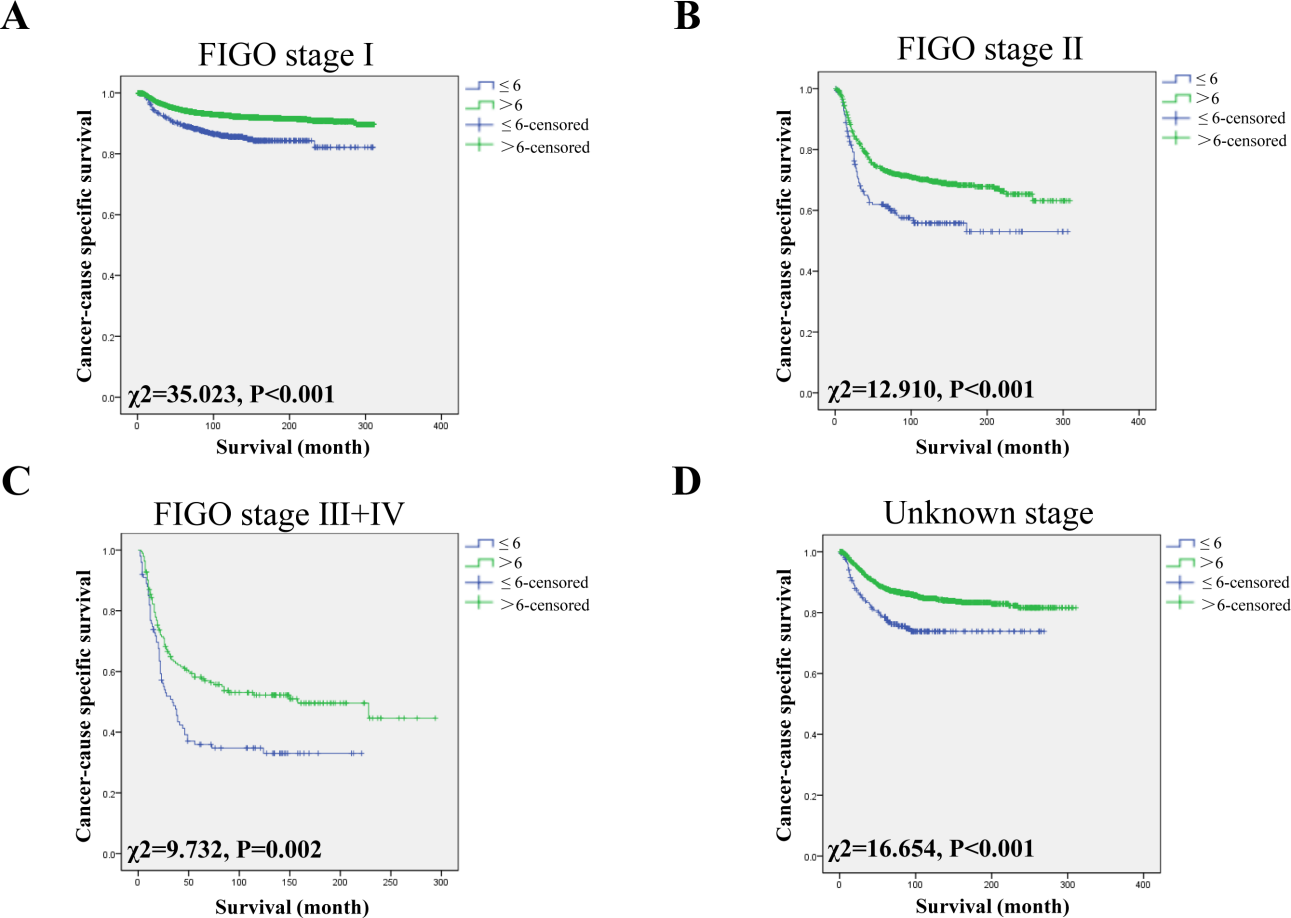
Log-rank tests of CSS comparing patients with NLNs (≤6 VS >6) for (**A**) FIGO stage I: χ^2^ = 35.023, *P* < 0.001; (**B**) FIGO stage II: χ^2^ = 12.910, *P* < 0.001; and (**C**) FIGO stage III + IV: χ^2^ = 9.732, *P* = 0.002 and (**D**) Unknown stage: χ^2^ = 16.654, *P* < 0.001.

According to the FIGO staging system, patients from SEER-data were divided into 5 subgroups, including stage I, II, III, IV and unknown. Since minimal cases in FIGO III and IV, we combined stage III and IV in one group: FIGO III+IV. We then further analyzed the effects of NLN on survival of each subgroup. We confirmed that the NLN count was an independently prognostic factor in each subgroup using univariate analysis (all *P <* 0.05) (Figure 2). After adjusting variables, the NLN count was also validated as an independent survival factor in FIGO stage I (NLNs >6, HR: 1.685, 95% CI: 1.338~2.122; *P* < 0.001), FIGO stage II (NLNs >6, HR: 1.512, 95% CI: 1.163~1.965; *P* = 0.002), FIGO stage III + IV (NLNs >6, 1.608, 95% CI: 1.154~2.242; *P* = 0.005) and unknown stage (NLNs >6, HR: 1.438, 95% CI: 1.050~1.971; *P* = 0.024) (Table 4).

**Table 4:** Univariate and multivariate analysis of NLN status on CSS of cervical cancer based on different cancer stage

Parameter	NLN	3-year CCS	5-year CCS	Univariate analysis		Multivariate analysis	
				Log rank χ^2^ test	*P*	HR(95%CI)	*P*
FIGO stage							
Stage I				35.023	0.000		0.000
	0~6	89.0%	83.2%			Ref	
	7~	92.7%	88..5%			1.685 (1.338~2.122)	0.000
Stage II				12.910	0.000		0.002
	0~6	60.3%	54.9%			Ref	
	7~	76.7%	66.9%			1.512 (1.163~1.965)	0.002
Stage III + IV				9.732	0.002		0.005
	0~6	46.0%	33.0%			Ref	
	7~	58.5%	51.3%			1.608 (1.154~2.242)	0.005
Unknown stage				16.654	0.000		0.024
	0~6	76.8%	70.0%			Ref	
	7~	87.1%	80.4%			1.438 (1.050~1.971)	0.024

*P*-values refer to comparisons between two groups and were adjusted for age, race, grade, histologic type, FIGO stage and radiation after surgery as covariates.

## DISCUSSION

Despite the current UICC/AJCC (Union for International Cancer Control /American Joint Committee on Cancer) and FIGO staging system do not consider the status of lymph nodes in cervical cancer, various studies have confirmed that it plays an important role in prognostic survival of patients [16–19]. Zhou *et al.* concluded that positive LN counts had prognostic value in lymph node positive stage I-II of cervical cancer [6]. However, the lymph node ratio and the number of positive lymph node were affected by many factors such as the number of lymph node retrieved and inspected, and neoadjuvant therapy. If the LN retrieved was not enough, the prediction of survival would be inaccurate. It has been reported that the NLN count was an independent prognosis factor in colon [20, 21], gastric [22], esophageal [14] and so on. Although Chen *et al.* [11] confirmed that the combination of NLNs count and the ratio of positive and removed lymph nodes could better predict the postoperative survival in patients with cervical cancer, the association between NLN counts and survival was not fully explored. In this study, we found that the rate of 5-year CCS of cervical cancer patients was improved with the increase of NLN count from 0 to 23 (all *P <* 0.001), and identified the optimal cutoff value for NLN count as 6. Furthermore, the NLN count was an independent prognosis factor for patients with cervical cancer of each FIGO stage. Apparently, NLN count is a good supplement for evaluation prognosis of FIGO stage.

Until now, there is no conclusion of mechanism of NLNs effecting on the prognosis of cervical cancer. Heeren *et al.* identified that an increased number of regulatory T cells (Treg) and the decreased CD8+ T cell/Treg ratio were found at both positive and negative lymph nodes in the regional lymph node area of cervical cancer patients, reflecting an immune suppressive microenvironment that promotes metastatic spread [23]. On the other hand, lymphatic micrometastasis is important to the prognosis of cervical cancer. Since it was difficult to find lymphatic micrometastasis during operation, we have to retrieve more NLNs to reduce the residual micrometastases and improve the prognosis of cervical cancer, which was consistent to our results.

Extent of lymphadenectomy is a matter of debate for cervical cancer treatment. Various studies have examined whether the number of retrieved lymph nodes would affect survival of cervical cancer patients. Lim *et al.* [24] found that the number of retrieved lymph nodes was an independent prognostic factor for cervical cancer treatment in bulky cervical cancer group. In this group, more than 40 lymph nodes had a significant positive effect on disease-free survival and overall survival using multivariate analysis. However, the number of retrieved lymph nodes was not an independent prognostic factor in the non-bulky cervical cancer group. Mao *et al.* [25] showed that if a standardized lymphadenectomy was performed, the number of lymph nodes removed was not an independent prognostic factor for patients with node-negative early cervical cancer. Zhou *et al.* [18] found that the number of positive lymph nodes had prognostic value in cervical squamous cell carcinoma and adenosquamous carcinoma, but not in cervical adenocarcinoma. Also, the number of positive lymph nodes is an independent risk factor for CSS of cervical cancer patients. In addition, Garg *et al*. [26] and Srisomboon *et al.* [27] explored the impact of different treatments on the prognosis of early cervical cancer. But no relationship between NLN count and prognosis was found in the setting of cervical cancer previously. Lymphatic micrometastasis plays an important role in the prognosis of cervical cancer. Since it was difficult to find lymphatic micrometastasis during operation, more NLNs have to be retrieved to reduce the residual micrometastases to improve the prognosis of cervical cancer. Thus, NLN count may perform better than total lymph node count or positive lymph node for prediction of survival.

The relationship between lymph node count and outcome was controversial in the treatment of cervical cancer. Because the majority of patients with early stage cervical cancer do not have lymph node metastases. The extensive lymphadenectomy was unnecessary and could cause complications such as lymphedema [25]. Pieterse *et al.* [28] concluded that more lymph nodes retrieved is related to longer survivals of patients with positive nodes. Similar results were found in the studies of Shah *et al.* [19] and Lim *et al.* [24], which concluded that early-stage cervical cancer patients who underwent a more extensive lymphadenectomy had longer survivals. In contrast, Ditto *et al.* [17] revealed that the number of lymph nodes had no effect on survival. The node-positive patients with cervical cancer are heterogeneous and the prognosis of these patients cannot be stratified by the definite node-stage. Thus, the concept of NLN count may serve as a prognostic indicator in various cancers.

This study has several limitations. Firstly, different operative approaches, doctors and even pathologist would affect lymph nodes harvest, which cannot be adjusted in analysis. Secondly, the SEER database does not have the information of subsequent therapy including adjuvant chemotherapy, radiotherapy and targeted therapy, co-morbidities and recurrence, which may also impact patients’ survival outcome. Thirdly, the SEER database lacks detailed description of preoperative clinical grading and response to treatment. Despite these potential limitations, this study demonstrated that the NLN count was an independent prognostic predictor for patients with cervical cancer. And 6 was identified as the optimal cutoff value of NLN count to divide patients into high and low risk subsets in terms of CSS. NLNs count could be a good supplement for evaluating prognosis of UICC/AJCC and FIGO stage of cervical cancer.

## MATERIALS AND METHODS

### Patient selection

The SEER database and SEER-stat software (SEER*Stat 8.3.2) were used to search cervical cancer with M0 after radical surgery between 2004 and 2012 with a known age (≥18). Years of diagnosis, age, race, grade, primary site, FIGO stage and CSS were extracted from the SEER database. Histological types were limited to squamous cell carcinoma (8070/3), adenocarcinoma (8140/3) and mucinous adenocarcinoma (8141/3, 8142/3). Survival time was calculated from the date of diagnosis to the date of cancer-specific death. The exclusion criterions included: age<18, receiving neoadjuvant therapy, no evaluation of histological type, multiple malignant neoplasms, died within 30 days or information on CSS and survival months unavailable.

### Statistical analysis

The NLNs cutoff points were determined using the X-tile program (http://www.tissuearray.org/rimmlab/), which identified the cutoff value with the minimum *P* values from log-rank χ^2^ statistics for the categorical NLNs in terms of CSS.

The X-tile plot illustrates the presence of substantial tumor subpopulations and shows the robustness of the relationship between a biomarker and outcome by construction of a two dimensional projection of every possible subpopulation [29]. In our data, the variables, negative lymph node count and cancer specific survival were used. X-tile plots provide a single, global assessment of every possible way of dividing a population into low/high or low/medium/high level marker expressions. X-tile data are presented in a right grid where each point represents a different cut-point. The intensity of the color of each cutoff point represents the strength of the association. The X-tile software allows the user to move a cursor across the grid and provides an “on-the-fly” histogram of the resulting population subsets along with an associated Kaplan-Meier curve [30]. Baseline characteristics were compared using the χ^2^ test for nominal variables. Survival curves were generated using Kaplan-Meier analyses, and the differences between the curves were analyzed by log-rank test. Cox regression models were built for analysis of risk factors for survival outcomes. Statistical analyses were performed using the statistical software package SPSS for Windows, version 19.0 (SPSS Inc., Chicago, IL). All *P* values were two-sided. *P <* 0.05 was considered statistically significant.
